# New treatment methods for myocardial infarction

**DOI:** 10.3389/fcvm.2023.1251669

**Published:** 2023-09-28

**Authors:** Bingbing Sun, Long Wang, Wenmin Guo, Shixuan Chen, Yujie Ma, Dongwei Wang

**Affiliations:** ^1^ Department of Critical Care Medicine, The Air Force Characteristic Medical Center, Air Force Medical University, Beijing, China; ^2^ Department of General Internal Medicine, Beijing Dawanglu Emergency Hospital, Beijing, China; ^3^ Wenzhou Institute, University of Chinese Academy of Sciences, Wenzhou, China; ^4^ Department of Cardiac Rehabilitation, Zhengzhou Central Hospital affiliated to Zhengzhou University, Zhengzhou, China

**Keywords:** coronary heart disease, myocardial infarction, cardiac regenerative medicine, scaffold, exosome

## Abstract

For a long time, cardiovascular clinicians have focused their research on coronary atherosclerotic cardiovascular disease and acute myocardial infarction due to their high morbidity, high mortality, high disability rate, and limited treatment options. Despite the continuous optimization of the therapeutic methods and pharmacological therapies for myocardial ischemia–reperfusion, the incidence rate of heart failure continues to increase year by year. This situation is speculated to be caused by the current therapies, such as reperfusion therapy after ischemic injury, drugs, rehabilitation, and other traditional treatments, that do not directly target the infarcted myocardium. Consequently, these therapies cannot fundamentally solve the problems of myocardial pathological remodeling and the reduction of cardiac function after myocardial infarction, allowing for the progression of heart failure after myocardial infarction. Coupled with the decline in mortality caused by acute myocardial infarction in recent years, this combination leads to an increase in the incidence of heart failure. As a new promising therapy rising at the beginning of the twenty-first century, cardiac regenerative medicine provides a new choice and hope for the recovery of cardiac function and the prevention and treatment of heart failure after myocardial infarction. In the past two decades, regeneration engineering researchers have explored and summarized the elements, such as cells, scaffolds, and cytokines, required for myocardial regeneration from all aspects and various levels day and night, paving the way for our later scholars to carry out relevant research and also putting forward the current problems and directions for us. Here, we describe the advantages and challenges of cardiac tissue engineering, a contemporary innovative therapy after myocardial infarction, to provide a reference for clinical treatment.

## 1. Background

In 2020, the World Health Statistics reported that cardiovascular disease ranked first among all non-communicable diseases in 2016, significantly higher than cancer ranking second (43.66% vs. 21.95%). The statistics revealed that, from 2002 to 2016, the mortality of myocardial infarction in China showed an upward trend of up to 42.23%–62.72% (1). Myocardial infarction, which is a subclinical classification of coronary heart disease with high mortality and disability rates, has been the focus of cardiovascular physicians for many years. This disease mainly involves acute and subacute events, such as subintimal hemorrhage, plaque rupture, plaque erosion, platelet and fibrin agglutination at the damaged part, thrombosis, coronary artery spasm, and small distal vessel embolism due to coronary atherosclerosis, leading to complete occlusion of the coronary artery lumen and complete cessation of blood supply. Consequently, this eventually causes myocardial necrosis of the ventricular wall in the blood supply area. Although the development and progress of percutaneous coronary intervention (PCI) and new thrombolytic drugs in cardiology have saved the lives of a large number of patients with acute myocardial infarction, the incidence rate of heart failure continues to increase year by year. Here, we describe the advantages and challenges of cardiac tissue engineering, a contemporary innovative therapy after myocardial infarction, to provide a reference for clinical treatment.

## 2. The rise of cardiac regenerative medicine

Human cells are divided into three kinds, namely, unstable, stable, and permanent cells, based on their capacity to regenerate. For a long time, it has been generally believed that striated muscle cells have weak regeneration ability and little significance for repair after injury. These cells are repaired by scarring. Surprisingly, after nearly 20 years of research, cardiac regenerative medicine has challenged this viewpoint. In the twenty-first century, research found that the expression of Ki-67 was detected in 4% of the infarct area and 1% of the myocyte nuclei outside the infarct area. Moreover, Ki-67 was reported as significantly related to cell division. Further, researchers proposed the regenerative potential of cardiomyocytes (CMs) and its possible key component in the increase of myocardial muscle mass (2). However, the self-regeneration rate of CMs remained <1% per year, which is unlikely to play an effective role in the repair of myocardial infarction (3).

With the development of regenerative repair medicine in recent years, cardiac tissue regeneration engineering has gained increasing attention through research and application of cardiac tissue regeneration materials and methods that promote cardiac repair and functional recovery after myocardial infarction, hence preventing the occurrence and development of heart failure. Cardiomyocyte regeneration therapy, a treatment primarily targeting the infarcted myocardium, promotes the regeneration of myocardial and vascular tissues in the infarct area by using various cells and biomimetic materials, to stimulate the recovery of cardiac mechanical function and electrophysiological activities and prevent the occurrence of cardiac remodeling and heart failure. With the in-depth study of cardiac regenerative medicine, the viewpoint of non-renewable CMs has been challenged repeatedly. What's more, researchers not only confirmed the existence of regenerated CMs after infarction from various types of experiments but also clarified the possible mechanism of regeneration from the aspects of molecular biology, cell metabolism, and gene regulation. However, compared with adult mammals, newborn mammals have a far stronger regeneration ability. For example, Porrello et al. (4) found that mice with partial resection of the left ventricular apical region on the first day of birth gradually achieved complete regeneration and repair of the injured myocardium for 21 consecutive days. Unfortunately, they confirmed that this regenerative ability declined on the 7th day of life. Similarly, Ye et al. (5) confirmed in the myocardial infarction model of large pigs that the ability of myocardial regeneration and myocardial remodeling on the second day of birth was better than that on the 3rd and 14th days. It was evident that the cardiac function of the infarction group on the second day of birth also improved. Similar studies (6–8) on animals and humans (9) have also been made. In general, these studies have shown that the mammalian heart also has transient regeneration ability.

**Figure 1:**
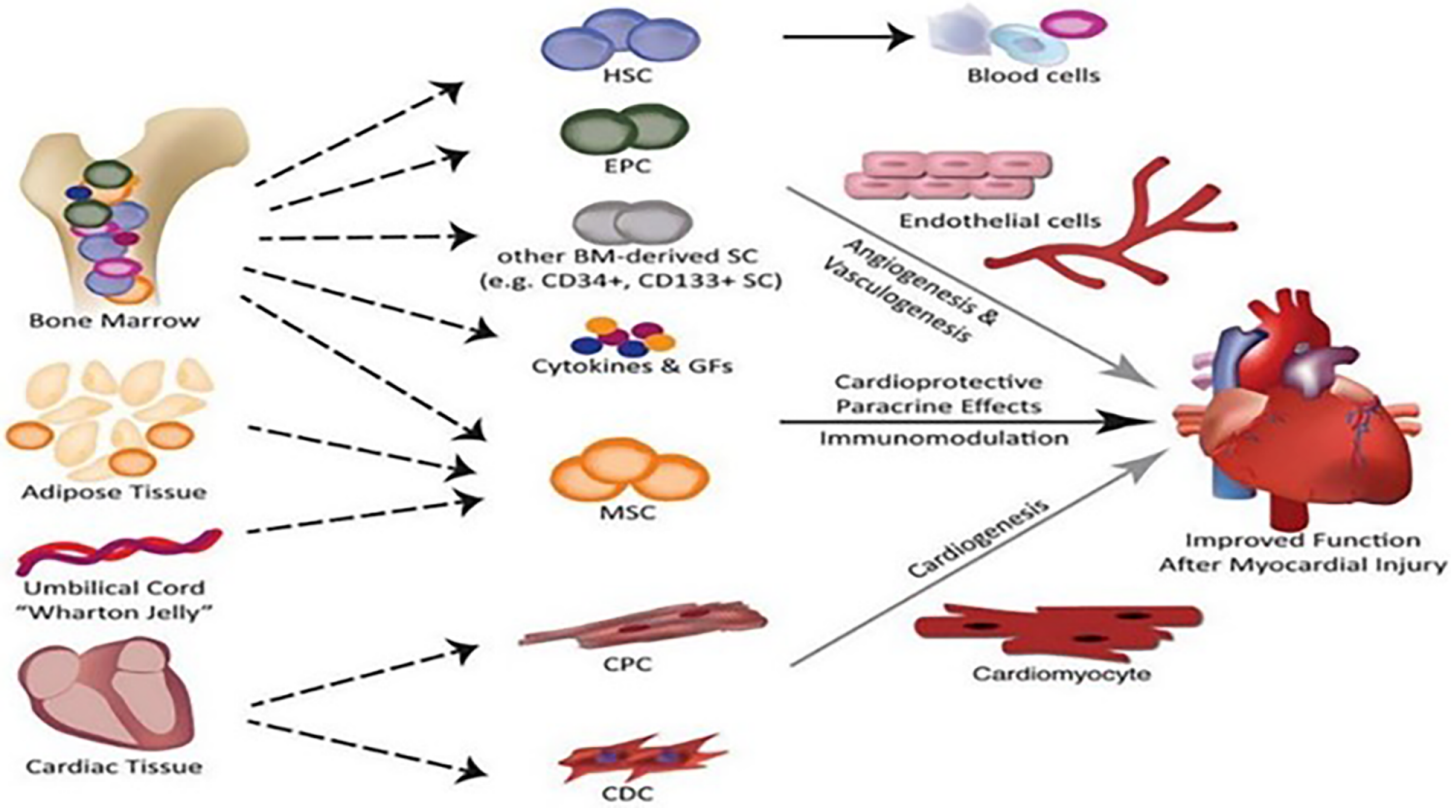
Schematic illustration of the cell types that may be used in cardiac regeneration therapy at present [adapted from Nguyen et al. (10)].

As a new medicine, regenerative medicine involves multidisciplinary participation and interaction. The realization of regeneration mainly includes three elements, namely, cells, scaffolds, and signal molecules. Specifically speaking, CMs, vascular endothelial cells (ECs), and smooth muscle cells (SMCs) are induced by various types of stem cells and progenitor cells, and cell sheets are formed by the combination of various cells. Scaffolds with bioactivity and mechanical properties are formed by processing various natural and synthetic materials. Extracellular bodies and paracrine active factors are produced by mesenchymal stem cells (MSCs), adipose stem cells, and inflammatory cells. Up to now, regenerative medicine has achieved initial results through various *in vivo* and *in vitro* studies and is expected to become another treatment option for patients with myocardial infarction. Figure 1 presents a schematic illustration showing the cell types that may be used in cardiac regeneration therapy at present [adapted from Nguyen et al. (10)].

## 3. Cells in myocardial regeneration

The ideal cell that meets the standard of cardiac tissue regeneration and repair should be an autologous cell with good control and clonality. Moreover, it must be able to differentiate into different cardiovascular cell lines. Long-term implantation and tissue regeneration are expected after the implantation of cells to the damaged myocardium. As a cell with unlimited differentiation and self-cloning potential, stem/progenitor cells can produce one or more progeny cells, attracting the interest of many researchers. Various types of stem cells, such as hematopoietic stem cells (HSCs), embryonic stem cells (ESCs), MSCs, induced pluripotent stem cells (iPSCs), skeletal myoblasts, and endothelial progenitor cells, have been studied as candidate cells in promoting myocardial repair after myocardial infarction. However, the application of some stem/progenitor cells in cardiac tissue regeneration is limited due to factors such as clinical safety, effectiveness, and medical ethics. In the following sections, we discuss the several types of stem/progenitor cells that have gained consensus.

### 3.1. Mesenchymal stem cells

MSCs are a type of adherent pluripotent cells that can express a variety of cytokines [such as stem cell factor (SCF), interleukin (IL)-1, and interleukin (IL)-6)], which can be derived from fibroblast-like cells in the bone marrow, adipose tissues, and others and can differentiate into specific cells such as adipocytes, osteoblasts, and chondrocytes (11). MSCs can rarely differentiate into functional CMs in a normal physiological environment and mainly promote the repair of cardiac injury through paracrine rather than direct myocardial differentiation. For example, Kinnaird et al. (12) demonstrated that MSCs promote the formation of arteries *in vitro* and *in vivo* by releasing vascular endothelial growth factor (VEGF) and fibroblast growth factor (FGF) to improve myocardial perfusion and reduce tissue ischemia. Through their *in vitro* and *in vivo* studies, Gnecchi et al. (13). and Mangi et al. (14)., respectively, found that the survival gene Akt1 produced by the bone marrow MSCs through overexpression and paracrine pathways played a protective role against ischemic injury, apoptosis, and necrosis of the co-cultivated CMs. In addition, MSCs not only promote cardiac repair through the paracrine effect but also exert their immunomodulatory properties through direct or indirect interaction with immune cells (15). The persistence of chronic inflammatory response is well known to play an important adverse role in ventricular remodeling in patients with ischemic cardiomyopathy (16). Regulating the process of inflammatory response after myocardial infarction may play a positive role in promoting the repair of myocardial injury after myocardial infarction. Luger et al. (17) found that the MSCs could weaken the response of neutrophils and natural killer cells in the infarct area by injecting them into the myocardial infarction mice model. A significant increase in the left ventricular ejection fraction was observed 4 weeks after myocardial infarction, confirming the key role of MSC immune regulation in cardiac repair. In addition, extracellular vesicles (EVs) secreted by MSCs also play an important role in cardiac tissue regeneration after myocardial infarction (18). These EVs were initially regarded as useless and ignored, but recently, many studies confirmed that they can induce a variety of signal cascades in recipient cells after releasing their lumen contents in suit by wrapping and protecting a variety of bioactive molecules. Recently, many studies showed that MSC-derived EVs play an effective role in reducing the infarct area, regulating the local inflammatory response of infarction, and improving heart function. EVs are described in detail below. Based on the above, MSCs are considered promising candidate cells for cardiac tissue injury repair.

### 3.2. Induced pluripotent stem cells

Inspired by the research of human ESCs, researchers began to focus on induced iPSCs. Takahashi et al. (19) won the Nobel Prize technology for solving the ethical differences of human ESCs, that is, by introducing embryonic elements into somatic cells, they accomplished the direct generation of pluripotent stem cells from somatic cells. *In vitro* studies have shown that iPSCs also have the multi-differentiation potential of stem cells according to their electrophysiological characteristics (20, 21). However, the initially generated iPSC-derived CMs (iPSC-CMs) are immature and imperfect cells with no organized sarcomere stripes and no expression of related functional genes [such as the ryanodine receptor (RyR2)] (22). Horikosh et al. (23). made the iPSC-CMs mature and established an effective strategy for the maturity and application of these cells by cultivating them in a three-dimensional environment or fatty acids and applying physical and mechanical stimulation. In 2014, Ye et al. (24) found that after injecting a mixture of iPSC-derived cells with CMs, SMCs, and ECs into a porcine model of acute myocardial infarction for 4 weeks, not only the cell mixture integrated into the host myocardium to improve cardiac function, but also no ventricular arrhythmia was found. This study established the safety of human iPSC therapy in cardiac repair. In addition, Zhao et al. (25) found that the beneficial effects of cell therapy are associated with paracrine mechanisms. Subsequently, Gao et al. (26) implanted a large patch composed of this kind of mixed cells in a porcine model of myocardial infarction and found not only a significant improvement in cardiac function and left ventricular remodeling but also the absence of arrhythmia. In 2018, Japanese researchers began the first clinical trial by transplanting an iPSC-derived cardiomyocyte mat into three patients with congestive heart failure. Unfortunately, no findings were reported about this trial (27). However, other researchers claimed that undifferentiated iPSCs posed a risk of potential teratoma and arrhythmia (28, 29) and that long-term culture would lead to genetic instability and karyotype abnormalities, implying that the heterogeneity of transplanted iPSCs must be limited to avoid possible immune rejection (30–32). Based on the abovementioned possible immune response and genetic instability, researchers are committed to establishing a genetically stable iPSC cell bank and developing a variety of immune tolerance strategies.

### 3.3. Cardiac progenitor cells

Adult stem cells have a limited myocardial differentiation potential, but resident cardiac progenitor cells (CPCs) derived from the heart may be “preprogrammed” into CMs. In particular, the discovery of rare CPCs with the ability to regenerate CMs paved the way for further research on CPCs in recent years (33–35). However, due to its low differentiation efficiency, it cannot meet the needs of regeneration after tens of thousands of myocardial infarction. Goumans et al. (36) improved the differentiation efficiency of myocardial progenitor cells through an *in vitro* TGF-*β*1 induction test, laying the groundwork for the subsequent application of various growth factors, microRNA, and drugs in the differentiation process of myocardial progenitor cells. Subsequently, Matsuura et al. (37) compared the repair effects of CPCs and adipose-derived mesenchymal cells on myocardial infarction in a mouse model. The result showed that the former was significantly better than the latter in terms of reducing the myocardial infarction area and improving cardiac function after myocardial infarction while also pointing out the possible mechanism of the secretion of various growth factors and the regulation of cellular signal pathways of CPCs. In addition, the new type of cardiac stem cells found in the human left atrium could also differentiate into cardiac phenotypes and express specific cardiomyocyte markers (38). Subsequently, Fanton et al. (39) transplanted the CMs induced by the left atrial stem cells in a miniature pig model of myocardial infarction and found that the left ventricular ejection fraction increased and the myocardial scar area decreased after transplantation. Moreover, during the subsequent 2-month follow-up, the transplantation increased the left ventricular ejection fraction, reduced the scar size, and improved the cardiac function of the miniature pigs. Later *in vitro* studies pointed out that CMs induced by the left atrial stem cells could promote angiogenesis through paracrine mechanisms (40). Although the clinical research has not yet started, preclinical research has proven that there is no arrhythmia, tumor, or other phenomena in animal model transplantation, which is regarded as a safe treatment (39). Other researchers found that in the context of post-myocardial infarction, the probability of CPCs or c-kit positive cells differentiating into CMs was extremely low and that the clinical significance of endogenous myocardial regeneration through CPCs was limited. A piece of evidence suggested that even when CPCs were injected into the infarcted myocardium, they may not differentiate into CMs, highlighting the need for caution in their clinical application (41).

In conclusion, each of the abovementioned cell types used for cardiac regeneration and repair has its pros and cons; in other words, there are several options for the application of cells in cardiac regeneration. However, due to the influence of ischemia and hypoxia at the infarct site and various inflammatory reactions, the low retention rate and low survival rate of implanted cells remain the key challenges affecting cell therapy. Cell therapy using CPCs and MSCs has consistently demonstrated safety and therapeutic efficacy, as evidenced by the following early-phase clinical trials and meta-analyses (42–47). However, the documented therapeutic effects are modest, posing challenges in transitioning to late-phase clinical trials. The application of scaffolds and extracellular bodies provides a solution to this problem.

## 4. Growth factors, cytokines, and exosomes in cardiac tissue repair

**Figure 2:**
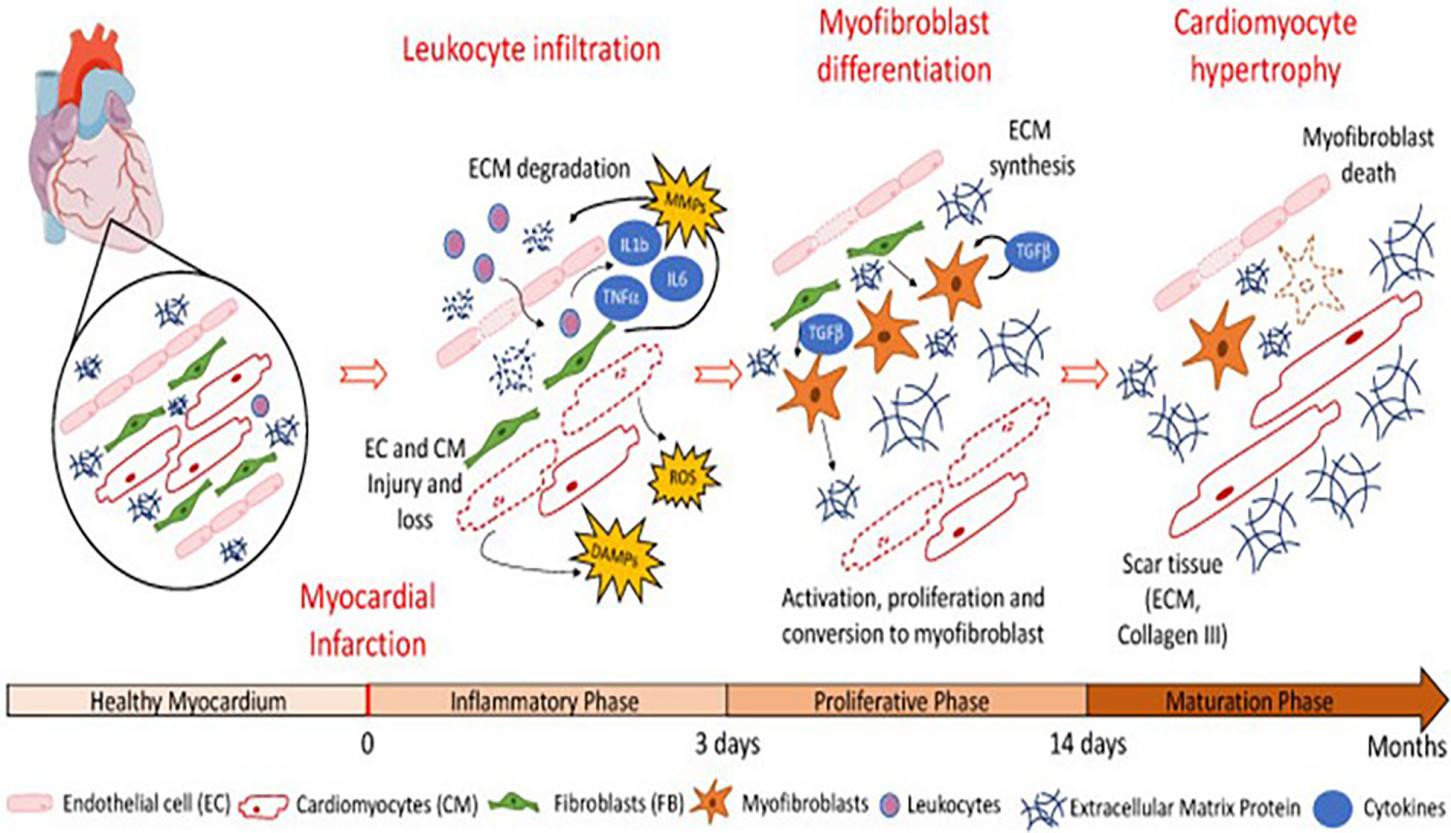
Schematic illustration showing that in the early stage of inflammatory response (0–3 days), various inflammatory factors (such as ROS, TNF-α, IL-6, IL-1b, and MMPs) are released and a large number of leukocytes infiltrated, resulting in the degradation of the extracellular matrix. In the proliferative stage, under the continuous action of chemokines and MMPs secreted by local fibroblasts, a large number of cardiac fibroblasts gather and stimulate the cicatricial repair. Finally, in the maturation phase, the necrotic myocardium in the infarct area is replaced by scar tissue [adapted from Riaud et al. (48)].

We all know that when a myocardial infarction occurs, an inflammatory reaction also follows. The pathological repair process of the heart after myocardial infarction mainly includes the inflammatory stage (0–3 days), proliferation stage (3–14 days), and mature stage (14 days–2 months). Figure 2 presents a schematic illustration showing that in the early stage of an inflammatory response (0–3 days), various inflammatory factors [such as reactive oxygen species (ROS), tumor necrosis factor (TNF)-α, IL-6, IL-1b, and matrix metalloproteinases (MMPs)] are released and a large number of leukocytes infiltrated, resulting in the degradation of the extracellular matrix (ECM). In the proliferative stage, under the continuous action of chemokines and MMPs secreted by local fibroblasts, a large number of cardiac fibroblasts gather and stimulate the cicatricial repair. Finally, in the maturation phase, the necrotic myocardium in the infarct area is replaced by scar tissue [adapted from Riaud et al. (48)]. Some researchers have even made a detailed analysis of the complex local inflammatory reaction process and the release of signal molecules in myocardial infarction (49–51). There is no doubt that the functional regeneration of CMs is inextricably linked to the interactions between cells and between cells and surrounding tissues and body fluids, in which the growth factors, cytokines, and exosomes play important roles.

### 4.1. Growth factors and cytokines for myocardial regeneration

With the in-depth study of cell therapy, an increasing number of researchers recognized that cells regulate tissue regeneration mainly through paracrine (52). As we all know, growth factors and cytokines play important roles in regulating the whole cell life cycle, including cell growth, proliferation, differentiation, and apoptosis. Naturally synthesized growth factors [e.g., VEGF, FGF, hepatocyte growth factor (HGF), and insulin-like growth factor (IGF)] and cytokines [e.g., IL, colony-stimulating factor, transforming growth factor-β family (TGF-β family), and TNF] cannot be directly used for treatment because of their inherent limitations as proteins produced in specific time and space. Researchers have synthesized growth factors and cytokines and combined them with today's advanced tissue engineering technology to develop their tremendous potential in myocardial regeneration therapy.

Firstly, the researchers optimize natural growth factors and cytokines through protein engineering to improve stability, high-speed blood clearance, short effective half-life, and uncontrolled explosive release. The majority of growth factors and cytokines mainly include the following aspects: regulating the protein stability and half-time (53, 54); improving the expression rate of key proteins (55); enhancing the affinity between protein and its receptor (56); and decreasing the internalization and recycling of protein receptors (57). Subsequently, they use engineered biomaterials including the natural polysaccharides and their analogs [e.g., chitosan (CHI), hyaluronic acid, and microbial polysaccharide] and biodegradable polymers (e.g., nanoparticle, hydrogel, microspheres, and layer-by-layer films) to further regulate the application of the abovementioned engineered proteins in regenerative therapy. For example, Feng et al. (58) injected the endogenous growth factors into mice subcutaneously with an injectable hydrogel. They found that the growth factors coated by hydrogel could promote angiogenesis *in situ*. In their study employing a rat model of myocardial ischemia–reperfusion injury, Sonnenberg et al. (54), using an ECM-derived hydrogel as the growth factor carrier, injected HGF carried by the hydrogel into the part of the myocardial injury. They found that the release time of HGF hydrogel was prolonged and the expression of genes related to myocardial fibrosis and angiogenesis was upregulated, which inhibited myocardial fibrosis, promoted angiogenesis, and improved the cardiac function of the rat. By using PLGA and sodium alginate, Choi et al. (59) synthesized a kind of microcapsules with a core–shell structure that could carry two growth factors and further regulate stem cell differentiation. Similarly, Azizian et al. (60) also used chitosan and gelatin to synthesize nanoparticles for an *in vitro* study of growth factors. In general, growth factors and cytokines have become popular and promising treatment options in the field of regenerative medicine (61).

### 4.2. Exosomes for myocardial regeneration

The exosome is a spherical membrane vesicle produced by paracellular secretion and mainly surrounded by a lipid bilayer, a subcellular structure released by cells under physiological and pathological conditions, and it can participate in the regulation of intercellular communication (62, 63). Research on exosomes found that not only they can be used as a container for various biological information in the physiological and pathological environment but also their internal components and microRNA content can be used as specific markers of cell activation and damage. Moreover, they can act as an intercellular messenger to participate in the interaction between cells (64). Therefore, it has attracted an increasing amount of attention. For example, in an experiment of a mouse model of myocardial infarction, Loyer et al. (65) found that exosomes with cytokines and growth factors (such as IL-6, chemokine ligands CCL2, and CCL7) were released by ischemic injured local myocardium and confirmed the same secretion phenomenon in human myocardial infarction. They speculated that these exosomes played an important role in regulating local response to injury and guiding cell migration. Other studies have also found that exosomes can not only directly interact with corresponding target cells in the form of receptors vs. ligands through their surface proteins and lipids but also they can indirectly play a local or remote regulatory role by releasing their internal signal molecules (such as matrix metalloproteinase, DNA, RNA, microRNA, and integrin) (66, 67). As is well known, a large number of platelets gather and activate in the infarct area, and exosomes derived from activated platelets inspire different signal transduction pathways in ECs, such as sarcoma (Src), PI3K, and extracellular signal-regulated kinase, resulting in the secretion of VEGF, basic fibroblast growth factor, and platelet-derived growth factor and finally promoting angiogenesis in the infarct area (68). Using a mouse model of ischemia–reperfusion injury, Arslan et al. (69) confirmed that mesenchymal stem cell-derived exosomes activated the PI3K/Akt pathway through glycolytic enzymes and antioxidant protein components, improved the level of local ATP and played an antioxidant role, and finally played a long-term regulatory role in the antioxidant injury. In addition, through the comparative study of iPSC and iPSC-derived EVs in a mouse model of ischemia–reperfusion injury, Pianezzi et al. (70) found that iPSC-derived exosomes carried molecular proteins that were conducive to cardiac regeneration and repair; resisted free radical injury; promoted the proliferation of CMs, vascular ECs, and SMCs; and finally promoted the repair of cardiac injury. Another study on exosomes derived from CPCs found that the latter could repair myocardial injury by inducing the aggregation and activation of endogenous cardiac stem cells at the site of myocardial infarction (71). There are many similar studies, and researchers have even compared the advantages and disadvantages and potential mechanisms of exosomes from different cell sources in promoting myocardial repair, such as exosome-induced autophagy (72, 73).

**Figure 3:**
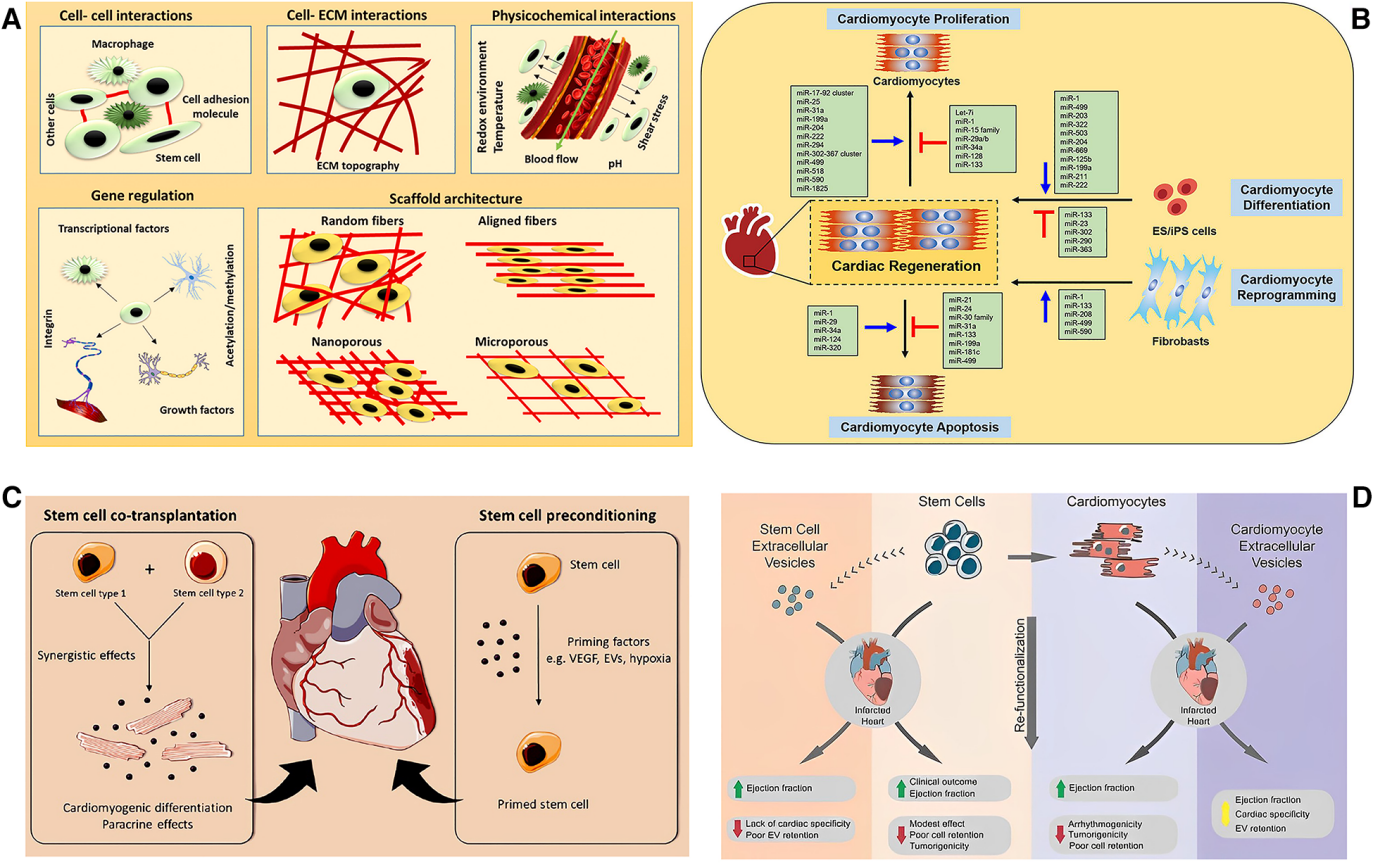
Schematic illustration of the application of exosomes. (**A**) The signal molecules/factors promote the change of stem cell niche in different ways to adapt to the stem cell function of the tissue in the stem cell microenvironment including cell–cell and cell–ECM interactions, physicochemical properties, gene regulation, and scaffold architecture [adapted from Augustine et al. (80)]. (**B**) Non-coding RNAs in cardiac regeneration [adapted from Yuan and Krishnan (81)]. (**C**) The combined transplantation of stem cells from two different sources can promote paracrine and induce cardiomyocyte differentiation, further promoting the repair of myocardial injury (left). The stem cells were pretreated with cytokines before transplantation to strengthen the functional activity of stem cells in promoting myocardial repair (right) [adapted from Beliën et al. (82)]. (**D**) The therapeutic potential and challenges of cell and extracellular vesicle-based therapies [adapted from Liu et al. (83)].

In addition to the abundant research of stem cell-derived exosomes, some researchers also hope to optimize the function of exosomes through integration or overexpression with new molecules, RNA fragments, or related proteins, to reduce the occurrence of off-target phenomenon and further improve the therapeutic effect of exosomes. In a study using a mouse model for myocardial infarction, Mackie et al. (74) confirmed that injecting CD34 + HSCs pretreated by Sonic Hedgehog (Shh) and the exosomes secreted from such stem cells into the peripheral area of myocardial infarction could not only enhance the vitality of CD34 + cells and induced angiogenesis in the infarct area but could also stimulate the activation of Shh signal pathway of other cell types through exosomes, significantly promoting angiogenesis and reduced myocardial infarction area. Yu et al. (75) injected exosomes secreted by bone marrow MSCs that overexpress the myocardial transcription regulator GATA4 into the site of myocardial infarction in rats. Such exosomes were found to enhance the anti-myocardial apoptosis effect of miRNA-19a and promote the repair of the ischemically damaged myocardium. In addition, some researchers modified the exosomes secreted by different stem cells with cardiac-targeting peptides (such as ctp-lamp2b and collagen hybridizing peptide) to improve the uptake of exosomes in the heart, better regulate the necrosis and apoptosis of local cells, eventually reduce the area of myocardial infarction, and improve cardiac function (76, 77). In addition, with the growing research on CMs, the role of the Hippo pathway in inhibiting the pathological proliferation of myocardium has gained increasing attention. For example, Leach et al. (78) and Liu et al. (79) discussed the relationship between the Hippo pathway and myocardial regeneration in detail and emphasized its effectiveness in preclinical trials conducted on pigs. All the abovementioned studies have confirmed that growth factors/cytokines and exosomes are promising treatment options for the recovery of cardiac function and cell differentiation and regeneration after myocardial infarction. Figure 3 presents a schematic illustration showing the application of exosomes:

a) The signal molecules/factors promote the change of stem cell niche in different ways to adapt to the stem cell function of the tissue in the stem cell microenvironment, including cell–cell and cell–ECM interactions, physicochemical properties, gene regulation, and scaffold architecture [adapted from Augustine et al. (80)].

b) The non-coding RNAs in cardiac regeneration (adapted from Yuan and Krishnan).

c) The combined transplantation of stem cells from two different sources can promote paracrine and induce cardiomyocyte differentiation, further promoting the repair of myocardial injury (left). The stem cells were pretreated with cytokines before transplantation to strengthen the functional activity of stem cells in promoting myocardial repair (right) [adapted from Beliën et al. (82)].

d) The therapeutic potential and challenges of cell and extracellular vesicle-based therapies [adapted from Liu et al. (83)].

## 5. Materials in cardiac tissue repair

Scaffolds play an important role as “bridge” and “link.” On the one hand, scaffolds provide a matrix and support for cell adhesion and proliferation. On the other hand, scaffolds can also regulate cell response in tissues with various signal molecules as clues and finally promote tissue regeneration and repair. It can be seen that scaffolds are crucial in a successful regenerative treatment.

**Figure 4:**
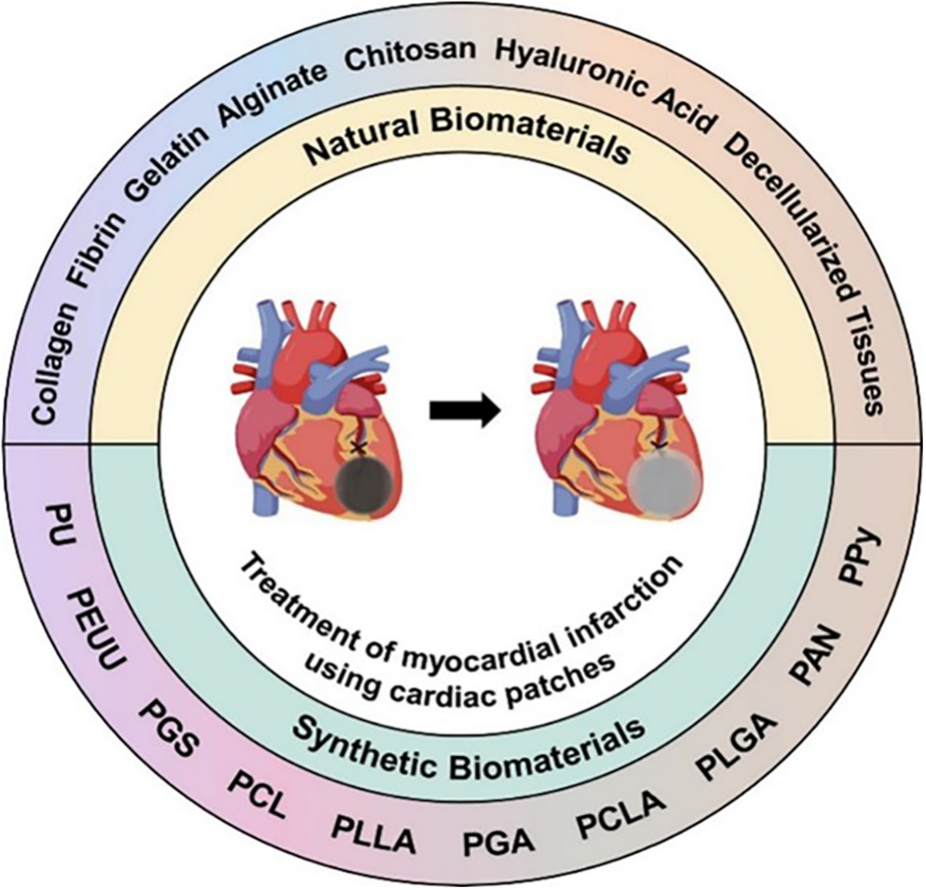
Schematic illustration of the types of scaffold materials currently used in cardiac regeneration therapy [adapted from Chang et al. (88)].

As an organ with continuous systole and diastole in the intrathoracic, the heart has high requirements for the mechanical properties of materials used for tissue repair after injury. Generally speaking, the materials used for cardiac scaffolds should have the following characteristics (84–87):

(a) The biocompatibility, that is, they must not cause collective immune rejection.

(b) The mechanical properties, that is, the material complies with the requirements of elasticity and mechanical properties during the contraction and relaxation of the heart.

(c) The porosity, that is, the scaffold should be porous, provide support for cells or bioactive factors, and act as a carrier to help them release to the damaged area, to enhance the therapeutic effect of cells. Previous studies have shown that the porosity of patches containing cells, which are conducive to the local survival of cells, is approximately 90% and the pore diameter is approximately 50 μm.

(d) The biodegradability, that is, both the material and its degradation products should be biodegradable, and the degradation rate should always be consistent with the formation of new tissue, to avoid the side effects attributed to prolonged existence in the body’s memory.

(e) The conductivity, that is, relating to the particularity of the heart as an organ coupled with electromechanical contraction. However, arrhythmia should be avoided. As far as the materials used for scaffolds are concerned, a wide range of choices can be used based on the abovementioned requirements. In terms of their sources, these materials can be roughly divided into two types, namely, natural materials (for instance, protein, polysaccharide, and acellular tissue) and synthetic materials [such as pyrolytic graphite sheet (PGS), polyurethane (PU), and polycaprolactone (PCL] (88). Figure 4 presents a schematic illustration showing the types of scaffold materials currently used in cardiac regeneration therapy [adapted from Chang et al. (88)]. In terms of their different uses, these materials can also be divided into conductive materials, super adhesive materials, stem cell delivery materials, drug delivery materials, and gene delivery materials.

### 5.1. Conductive materials

Generally speaking, conductive materials can be divided into three categories: synthetic conductive polymers [such as polypyrrole (PPy), polyaniline (PANi), and polythiophene (PTh)], carbon-based nanomaterials (e.g., carbon nanotube, graphene oxide, and reduced graphene oxide), and metal nanomaterials (e.g., gold, silver, and related alloy). For example, as a synthetic conductive polymer, polymer polypyrrole (PPy) has advantages such as superior biocompatibility, natural conductivity, no cytotoxicity, adjustable surface biochemical characteristics, and easy access (89, 90). All these eventually draw the attention of many researchers, making it one of the most widely studied conductive biomaterials. For instance, in the study of a mouse model of cryoablation myocardial injury, Cui et al. (91) proved that PPy and chitosan polymer could not only support the normal growth of CMs in the polymer but also promote the transmission of electrical pulses and shorten the QRS interval in the scar area through intramyocardial injection, thereby promoting the recovery of electrical conduction activity of injured myocardium. Similarly, He et al. (92) reached the same conclusion in the rat model of ischemic myocardial injury. In the study, they injected the PPy–chitosan hydrogel into the fibrotic scar area on the 7th day of myocardial infarction and tried different concentrations of the polymer. Finally, they found that the polymer could reduce the resistance between the tissues and improve the conductivity of the scar area, thus promoting the synchronous contraction of the heart. They also confirmed that the electrical conductivity increased proportionally with the addition of PPY–CHI hydrogels. In addition, PANi and PTh and its derivatives can play the same important role in restoring the electrical conductivity of damaged myocardium equivalent. For details, please refer to other relevant literature. In addition, as another conductive material, carbon nanotubes also draw the attention of generations of researchers on regenerative medicine because of their unique physical structure, mechanical properties, and electrical conductivity. For example, Shin et al. (93) used the biological ink made of Gel-MA/DNA-coated carbon nanotubes and hemagglutinin (HA)/DNA-coated carbon nanotubes for myocardial repair through 3D bioprinting. The results showed that the above carbon nanotube mixtures played important positive roles in promoting myocardial repair and synchronous electrical conduction and mechanical contraction. Pryzhkova et al. (94) found that carbon nanotubes could induce the differentiation of human pluripotent stem cells into each germ layer and that this differentiation was affected by the surface roughness, morphology, and stiffness of carbon nanotubes. Ren et al. (95) reached the same conclusion in the study of super-aligned carbon nanotubes. This potential can also be found in the relevant research on graphene, graphene oxide materials, and metal nanomaterials (96–98).

### 5.2. Super adhesive materials

For a long time, the low retention ratio and low adhesion rate of transplanted cells in the damaged part and auxiliary biomaterials are some of the key links that hinder cell therapy in regenerative medicine. The in-depth study of the properties and functions of different materials helps in solving this problem. As mentioned above, materials are mainly divided into natural and synthetic types. In terms of the performance of scaffolds, synthetic materials have advantages such as controllable physical and chemical properties and mechanical functions, but they often lack biocompatibility due to the insufficiency of cell adhesion sites. On the contrary, most natural materials have sufficient biocompatibility, but the mechanical stress is relatively poor. In the preparation of tissue engineering scaffolds, they are often combined to obtain composites with both advantages, namely, the super adhesive materials. For example, in an experiment of a frozen myocardial injury mouse model, Frati et al. (99) synthesized an IGF-1 functionalized 1,3-propanedio (PDO)-enhanced sodium alginate/gelatin sponge with super strong cardiomyocyte adhesion characteristics, which could not only increase the local retention of CMs in the infarcted region but could also provide mechanical and nutritional support for the damaged myocardium and inhibit left ventricular dilation, thereby promoting the recovery of myocardial injury. In addition, in recent years, the hydrogel has attracted increasing attention due to its minimally unique viscoelastic nature. In terms of the materials used for making them, hydrogels can be made by combining different materials such as natural materials and synthetic materials. For instance, in a rat model of ischemia–reperfusion injury, considering the advantages of the ECM as a natural cell adhesive, Wassenaar et al. (100) injected the ECM-based hydrogel into the injured myocardium, which significantly reduced the negative remodeling of the injured myocardium and improved the cardiac function. In another study, Hong et al. (101) used a UV-activated technology to synthesize a super adhesive matrix hydrogel, specifically the Gel-MA/HA-Niobium (NB) mixed hydrogel. In an experiment of a porcine model of myocardial injury and carotid artery injury, it was found that the strong adhesion of the hydrogel could not only quickly stop bleeding but also promote the rapid wet adhesion of peripheral cells, thus promoting the repair of cardiovascular damage.

### 5.3. Stem cell delivery materials

Stem cell therapy has long been the focus of myocardial regeneration therapy research. The low implantation rate and low survival rate of transplanted cells remain important challenges faced by this therapy. Given the abovementioned problems, researchers have made many explorations on stem cell delivery methods and delivery materials.

Firstly, Kobayashi et al. (102) made a double-layer dressing of adhesive mesenchymal stromal cells using collagen as a stem cell carrier. This significantly improved the cardiac function of mice with congestive heart failure by increasing the implantation of donor cells and upregulating the expression of genes related to myocardial tissue repair. In another study, Kim et al. (103) used an imprinting technology to make the polyvinyl alcohol/polyethylene glycol hydrogel and ECM into a stretchable ECM patch, which enhanced the local delivery of MSCs in myocardial infarction and significantly improved the cardiac function in mice. In addition, in recent years, metal nanoparticles, graphene, carbon nanotubes, inorganic nanoparticles, and other materials are also used for stem cell delivery. For example, Gelmi et al. (104) found that the stem cell scaffold made of polylactic acid glycolic acid fiber, which was coated with conductive PPy, could induce the differentiation and maturation of human-derived pluripotent stem cells. Similarly, Ryan et al. (105) confirmed that a mixture with a high concentration of graphene collagen could not only promote the differentiation of ESCs into CMs but could also stimulate the maturation and proliferation of myofibroblasts and differentiated CMs during the *in vitro* cell culture experiments, paving the way for future graphene related *in vivo* experiments.

### 5.4. Drug/gene delivery materials

With the growing research on myocardial tissue regeneration therapy and inspired by tumor-targeted therapy, myocardial drug-targeted therapy and gene-targeted therapy are also developing. The selection of drugs and gene delivery materials has become the key link in achieving the treatment goal. At present, nanoparticles made of various materials are mainly used for drug and gene delivery, e.g., liposome nanoparticles, cationic dendrimers, cyclodextrin polymers, polyethyleneimines and metal nanoparticles, other polyplexes, and even nanoparticles such as viruses and phages. The following is a brief overview of the experimental researches related to different delivery materials.

At present, small molecule drugs that may be used in the treatment of myocardial infarction include prostaglandins, prostaglandin E2, prostaglandin I2, pyrvinium pamoate, pyrvinium pamoate, and dipeptidylpeptidase IV. In terms of the small molecule drug delivery materials, Ishimaru et al. (106) used a collagen sheet to deliver ONO1301 into mice with dilated cardiomyopathy. The results showed that the expression of endogenous repair-related factors, such as HGF and stromal cell-derived factor 1, in the myocardium was upregulated in the collagen group and that the anti-heart failure effect was significantly stronger than that in the ONO1301 group. Nakamura et al. (107) synthesized a kind of nanoparticle consisting of PLGA and ONO1301 and injected it into the area of the ischemic myocardium of mice by intramyocardial administration, which not only reduced the side effects such as diarrhea caused by systemic administration but also significantly improved the cardiac function through the sustained-release effect of the microspheres. In addition, the same is true for gene delivery materials. Traditional viral or non-viral systematic delivery methods cannot meet the demands of regenerative medicine for high-level and sustained gene therapy. The delivery mode with scaffold as a carrier provides new hope for gene therapy. For example, in an experiment of an ischemia–reperfusion injury mouse model, Michael et al. (108) injected microRNA-29B loaded with hyaluronic acid hydrogel into the myocardium around the infarcted myocardium. They found that compared with the single-injection hydrogel group, the microRNA-29B group had lower myocardial fibrosis, increased angiogenesis, and better cardiac function than the other. In another study, Li et al. (109) found that microRNA could induce reprogramming of cardiac fibroblasts into CMs *in vivo* and *in vitro*, using three-dimensional tissue hydrogel as a carrier. In addition, Zhou et al. (110, 111) used electrospinning technology to manufacture a double-layer scaffold consisting of PCL/gelatin/poly(l-lactic acid-co-ε-caprolactone. They used double-layer scaffolds as carriers to make artificial blood vessels with microRNA-126 and human umbilical vein ECs on the surface and then implanted the blood vessels into the carotid artery of rabbits. The results showed that the nanofiber scaffolds could enhance the endothelialization effect regulated by microRNA-126. In addition, the ECM as a gene vector also has been reviewed (112, 113).

## 6. Combined application of different treatment methods in cardiac tissue repair

**Figure 5:**
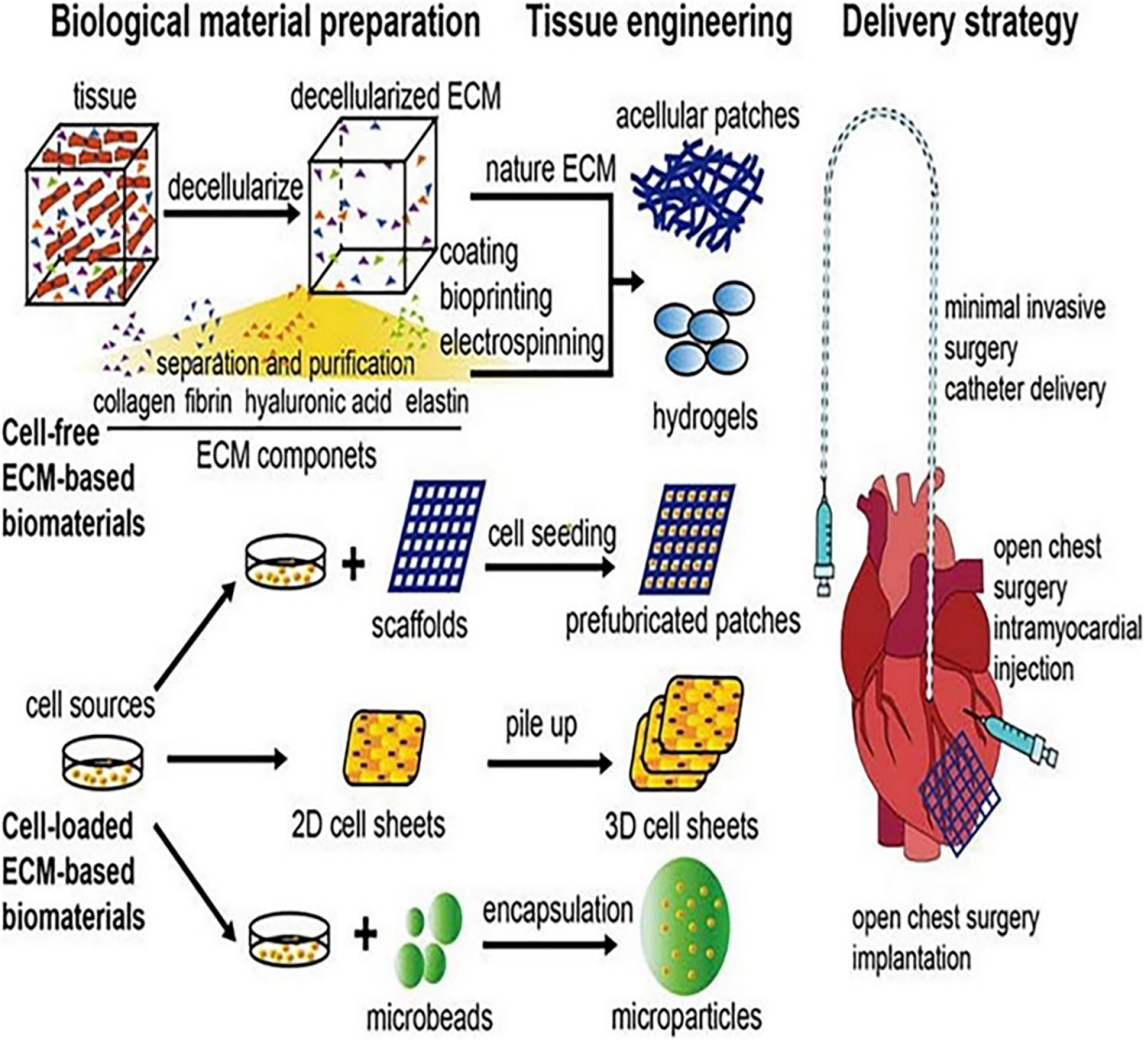
Schematic illustration of the combined application of scaffolds, cells, and extracellular signal molecules in cardiac regeneration therapy [adapted from Li et al. (114)].

In today's medicine, the cardiac tissue engineering strategy integrating cells, bioactive factors, and biomaterial scaffolds has increasingly shown broad prospects in cardiac repair and regeneration. Considering the complex process of cardiac tissue regeneration, it is agreed that the combined application of cells, scaffolds, and bioactive factors is necessary. Figure 5 presents a schematic illustration showing the combined application of scaffolds, cells, and extracellular signal molecules in cardiac regeneration therapy [adapted from Li et al. (114)]. For example, a study using poly(lactic–glycolic acid) (PLGA) as a microcarrier showed that compared with the transplantation of human-derived adipose-derived stem cells (ADSCs) alone, ADSCs with biomimetic surface functionalized PLGA as a microcarrier increased the survival and implantation of cells in the injured area after implantation in the rat model of chronic myocardial injury (115). That microcarriers were speculated to provide three-dimensional anchoring support for implanted cells and then stimulate the survival of transplanted cells. Similar studies have shown that the combination of human-induced pluripotent stem cell-derived CMs with poly-D-lysine and collagen-coated PLGA microcarriers can prolong the transplantation survival time of the former for up to 2 months (116). Other studies have shown that ADSCs based on injectable hydrogel can not only provide mechanical support for cells but also generate a variety of pro-angiogenic factors through ADSC paracrine *in vivo*, such as VEGF, HGF, and FGF-2, thus promoting angiogenesis and wound repair (117). In addition, the 0.05% Gel-MA-coated three-dimensional expanded nanofiber scaffold with hyper-elasticity and shape reversibility, which was manufactured by Chen et al. (118) and Qian et al. (119), greatly helped in the repair of heart, bone, nerve, and other injuries by loading a three-dimensional tissue structure with different cells, bioactive factors, and other components.

All of the abovementioned studies show that the combination of cells, carriers, and EVs can significantly improve the survival rate of cells and can also mediate the improvement of cardiac function through cell paracrine. It has become a promising direction in cardiac tissue engineering.

## 7. Challenges of cardiac regeneration

Although there are many studies on cardiac regenerative medicine, there are still many problems in the application of the above “road.”

### 7.1. How to accurately act on the target?

**Figure 6:**
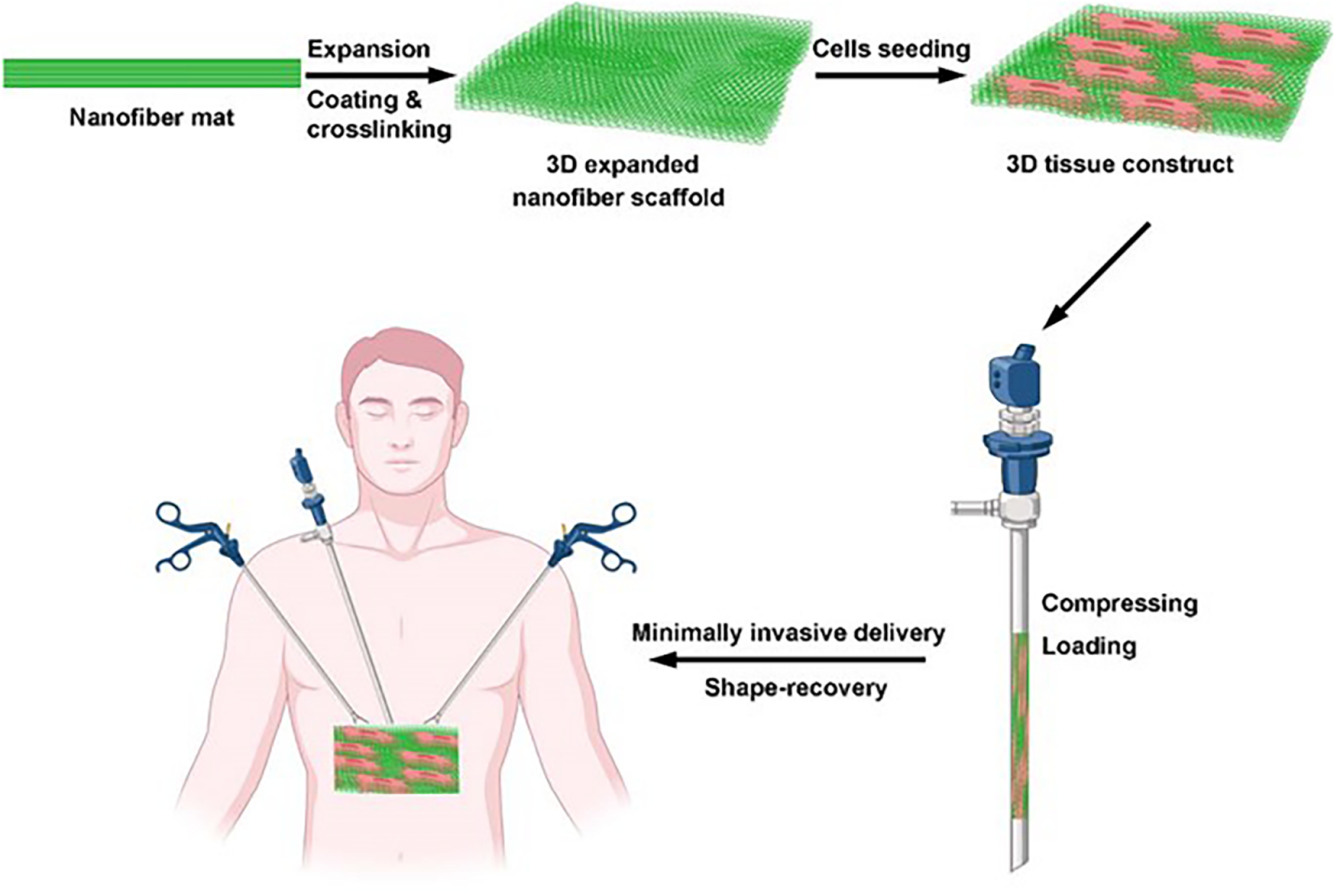
Schematic illustration of a hydrogel patch with a three-dimensional structure that can be transported to the body through a minimally invasive method and has a completely recoverable shape after transmission [adapted from Chen et al. (118)].

Just as people worry, off-target can not only play the expected role but can also have the risk of secondary ectopic tumors. In this regard, researchers have tried to inject cells into the veins, coronary artery, myocardium, epicardium, and pericardium. However, because of the low local survival rate, short survival time, and even the increased risk of local embolism and ectopic tumor, most of them failed. Therefore, other researchers focus on the application of heart patches to solve this problem. However, this kind of patch needs to be implanted into the injured area through a thoracotomy, which can cause large trauma, involves only a limited suitable population, and has a relatively high incidence of perioperative-related complications that are unfavorable to its clinical application. In response to these problems, researchers began to explore minimally invasive ways, such as the delivery of injectable hydrogel through an endoscope, and continuously make innovations in the delivery materials, such as mixing cells with hydrogels, encapsulated hydrogel beads, and microparticles/microspheres inoculated by cells. For example, by injecting a fibrin-supported three-dimensional stem cell spheroid tissue into the myocardium of rats with ischemia–reperfusion injury, Han et al. (120) discovered that the stem cells were induced by cardiomyocyte differentiation, then the infarct area was reduced, and finally the cardiac function was improved. By injecting CPCs or MSCs derived from exocrine hydrogel into the endocytic cavity of the toothed animal, Zhu et al. (121) firmed that the exocrine hydrogel could inhibit myocardial remodeling and promote cardiac function recovery after myocardial infarction. Through the subcutaneous injection of three-dimensional expandable hydrogel in a myocardial infarction rat model, Jiang et al. (122) pointed out that the hydrogel also could accelerate the adhesion, proliferation, differentiation, and angiogenesis of the injury even by subcutaneous injection. In recent years, many *in vivo* and *in vitro* studies have been published. Minimally invasive infusion. Figure 6 presents a schematic illustration showing a hydrogel patch with a three-dimensional structure that can be transported to the body through a minimally invasive method and has a completely recoverable shape after transmission [adapted from Chen et al. (118)], which avoids the trauma, complications, and other problems caused by thoracotomy. It is relatively simple and easy to operate. Although the relevant clinical significance requires a larger-scale sample of clinical experimental research, it remains a promising delivery method when compared with others.

### 7.2. When is the best time for regenerative intervention?

As previously stated, the pathological repair of cardiac injury after myocardial ischemia occurs in different stages, and each stage has its own different local reaction mechanisms. Although many studies on cells and scaffolds that are related to myocardial infarction repair have been published, there are still few studies on figuring out when is the best time for regenerative intervention. In a related study, Yoshizumi et al. (123) injected an absorbable chitosan hydrogel into the myocardium of rats at different time points after myocardial infarction (immediately after myocardial infarction, 3 days after myocardial infarction, and 2 weeks after myocardial infarction). They found the thickness of the ventricular wall and left ventricular function was improved at all injection time points. However, further comparison found that 3 days after myocardial infarction, the injection group exhibited a better functional outcome, better local vascularization, and fewer inflammatory markers compared with the other groups. Although the abovementioned research may suggest that 3 days after myocardial infarction is the best time of application, considering the differences and respective characteristics of human and animal models, the determination of the best delivery time still needs further large-scale and multicenter research.

Other problems, such as low cell retention and survival rate and lack of large-scale clinical research, are problems that must also be solved for the development of cardiac regenerative medicine.

## 8. Perspectives

In today's society, with the development of the economy and the transformation of bio-psycho-social medical models, people have raised higher requirements for the pursuit of health and quality of life. Translational medicine, also named translational research, as a new discipline rising in the 1990s, conforms to the requirements of this era (124). It emphasizes the core concept of patient-centeredness, follows the development idea of “bench to bedside” and “bedside to bench,” and attaches importance to the close combination of basic research and clinical treatment, to promote the effective transformation of the latest achievements of medical basic research in clinical practice and promote the development of medical applied science (125, 126). As previously discussed, myocardial infarction, heart failure, and other non-communicable diseases occur frequently in clinics. In the process of disease occurrence and development, they not only have their common characteristics (such as injury, remodeling, and dysfunction) but also have their personalized characteristics (such as acute attack stage and chronic remission stage). This complex situation requires us to connect traditional therapy with myocardial regenerative medicine research with the help of a translational medicine model. In other words, solving the problems endangering patients' lives by using PCI and coronary artery bypass grafting (CABG), delaying disease progress, and improving disease prognosis by using standardized drugs and rehabilitation treatment deepen the research and application of regenerative medicine and overcome the problems of regeneration and repair from the root, to stop further development of myocardial infarction and heart failure. In terms of the specific implementation of the transformation, I think, on the one hand, that it needs to be based on the mature and complete development of regeneration research, that is, regenerative medicine has solved many problems, such as the safety of implants, the functional survival of implanted cells, and the biological performance of implanted stents. On the other hand, it has been repeatedly confirmed and recognized by clinical studies at different stages. On this premise, traditional treatment and regenerative medical treatment can play a complementary and synergistic role. For example, the problem of implant delivery path can be solved by traditional means, such as PCI and CABG, and the problem of clinical inability or unwillingness to carry out reperfusion treatment can be solved by the application of regenerative means.

## 9. Conclusions

In conclusion, considering the complexity of the heart itself and cardiovascular diseases and the personalized characteristics of different patients, cardiac regeneration and repair treatment, as new treatment methods, are promising treatment options for preventing ventricular dilation after myocardial infarction, reducing cardiac remodeling, and improving cardiac function. However, how to design a safe, non-toxic, long-lasting, effective, simple, and easy treatment scheme and how to combine it with a clinic that is easy to be accepted by both doctors and patients have a long way to go.
